# Nasal staphylococci community of healthy pigs and pig-farmers in Aragon (Spain). Predominance and within-host resistome diversity in MRSA-CC398 and MSSA-CC9 lineages

**DOI:** 10.1016/j.onehlt.2023.100505

**Published:** 2023-02-11

**Authors:** Idris Nasir Abdullahi, Carmen Lozano, Carmen Simon, Javier Latorre-Fernandez, Myriam Zarazaga, Carmen Torres

**Affiliations:** a Area of Biochemistry and Molecular Biology, OneHealth-UR Research Group, University of La Rioja, Logroño, Spain; b Faculty of Veterinary Medicine, University of Zaragoza, Zaragoza, Spain

**Keywords:** Pigs and pig farmers, Nasal staphylococci, LA-MRSA, MRSA-CC398, Antimicrobial resistomes, Staphylococcal zoonosis, Coagulase-negative staphylococci

## Abstract

This study investigated the diversity and carriage rate of nasal *Staphylococcus* spp., and within-host variability of antimicrobial resistance (AMR), virulence determinants, immune evasion cluster (IEC) types and genetic lineages of *S. aureus* isolates. Also, the co-carriage rate of CoNS with *S. aureus* in the same nasal niche of healthy pigs and pig-farmers were studied in four pig-farms (A-D) in Aragon (Spain). Nasal samples of 40 pigs (10 pigs/farm) and 10 pig-farmers (2–3/farm) were collected for staphylococci recovery and isolates (up to 9 per sample) were identified by MALDI-TOF-MS. The virulence and AMR genes and *spa*-types of *S. aureus* isolates were investigated by PCR/sequencing. Of the 243 staphylococci identified (10 different species), 142 were *S. aureus* and 51 distinct isolates were selected for further characterization (that corresponded to one *S. aureus*/sample or more than one if they showed different AMR phenotypes). The highest carriage rate in pigs was *S. aureus* (65%) and *S. chromogenes* (22.5%), whereas in the pig-farmers, *S. aureus* (80%) and *S. epidermidis* (40%). Methicillin-resistant *S. aureus* (MRSA) were detected in 60% of pigs and 70% of pig-farmers. Only six *S. aureus* isolates were methicillin-susceptible (MSSA), all from farm-C. A multidrug resistance (MDR) phenotype was detected in all MRSA and in 83.3% of the MSSA isolates. All MRSA isolates were CC398 with *spa*-type t011 being the predominant (92.7%), while t034, t1451 (only in pig-farmers) and t4571 (in pigs) were also found. MSSA-CC9 isolates (t191, t1430) were detected in farm-C. All *S. aureus* isolates were negative for *luk-S/F-PV, tst,* and *scn* genes, except one MSSA-CC45-t065-IEC-type C isolate from a pig-farmer. About 34.6% and 75.0% of the pigs and pig-farmers *S. aureus* carriers, respectively, harboured within-host varied *spa*-types or resistomes. Moreover, 40% of pigs and pig-farmers with MRSA-CC398 had no CoNS nasal co-carriage, and 23.3% had ≥2 CoNS carriage. Conversely, only 16.7% of MSSA carriers had no CoNS co-carriage, whereas 50% had ≥2 CoNS carriages. The very high MRSA level and within-host resistome diversities highlight the need for multiple samplings to account for the dynamics of AMR crisis and control of inter-host transmission of *S. aureus* in pig-farms using “One Health” approach.

## 1 Introduction

Staphylococci are classified into two groups based on their ability to form fibrin (clot) in rabbit's plasma, viz: coagulase-positive staphylococci (CoPS) for species that do, and coagulase-negative staphylococci (CoNS) for species that do not [1]. CoPS are generally considered more pathogenic than CoNS [1]. The CoNS have recently elicited interest due to their increasing role in the incidence of opportunistic staphylococcal infections [2]. For instance, they have been associated with prosthetic joint infections or sepsis in immunocompromised patients, among others [3]. Whereas, *S. chromogenes* and *S. sciuri* have been isolated in exudative epidermitis cases in pigs [4,5]. Moreover, some CoNS carry mobile genetic elements (MGEs) that could be acquired by certain *S. aureus* strains via horizontal transfer [6]. For instance, some CoNS harbour *mecA* and *mecC* genes in SCC*mec* elements, thus considered potential reservoirs of AMR genes [6].

Among the CoPS, *S. aureus* is the most frequently detected, ubiquitous and has the greatest relevance in the One-Health ecosystems [7]. Other CoPS species specifically colonize certain groups of animals, these include; *S. pseudintermedius* (pets and horses), *S. intermedius* (pets)*, S. delphini* (horses)*, S. cornubiensis* (humans), *S. ursi* (black bears) and *S. coagulans* (dogs) [1,[8], [9], [10]]. In the livestock industries, an epidemiologically relevant strain often referred to as livestock-associated methicillin-resistant *S. aureus* (LA-MRSA)-CC398 is highly prevalent in European pig-farms and has been demonstrated to carry a multi-drug resistance phenotype of great relevance in human and veterinary medicine [11]. Most available epidemiological studies focused mainly on LA-MRSA of livestock and/or in-contact persons [11]. However, the prevalence and diversity of CoNS in pigs, pig-farmers and within-host variability of AMR genes and genetic lineages of *S. aureus* strains have not been very well established in Spain.

Livestock and humans are often colonized by a variety of CoNS. The CoNS are often commensals and their presence in any body part might inhibit the colonization of *S. aureus* [12]. For instance, it has been suggested that *S. epidermidis* may prevent *S. aureus* colonization in humans [13], whereas, the presence of *S. sciuri, S. saprophyticus* and *S. cohnii* are very rarely co-carried in combination with *S. aureus* in nares of pigs [14]. Essentially, some CoNS encode autoinducing peptides that could inhibit the *S. aureus* accessory gene regulator system [15]. This biochemical cross-talk between *S. aureus* and *S. epidermidis* has been suggested for the prevention of MRSA colonization [16].

Antimicrobial agents are often used in livestock production and their misuse in pig-farming has led to the emergence of AMR due to selective pressure on the microbiota exposed to these agents (as is the case of staphylococci) [17]. Aside from AMR to beta-lactams, a critically important one common with pigs' staphylococci is linezolid resistance (LZD^R^). Linezolid is an oxazolidone that has frequently been used as a last-resort antimicrobial agent against MRSA infections [18]. Hence, LZD^R^ is a high-priority phenomenon in clinical chemotherapy. This resistance is often associated with chloramphenicol, lincosamides, streptogramin A, and pleuromutilins resistance, often mediated by *cfr* gene [19]. Due to the long history of chloramphenicol usage in pig-farming, this agent has gradually lost its effectiveness as a result of the development of AMR by *S. aureus* [20]. This chloramphenicol resistance is often mediated by enzymatic inactivation (by *catA* and related genes as *cat*_*pC194*_*, cat*_*pC221*_*, cat*_*pC223*_), or efflux (by *fexA, fexB)* [20]. Some of these CLO^R^ genes are occasionally associated with LZD^R^ [19,20].

Livestock that are nasally colonized by *S. aureus* strains may directly spread them to humans or through the food chain (indirectly) [21]. Pigs are considered major hosts for zoonotic *S. aureus* transmission to humans [22]. *S. aureus*/MRSA has also shown economic importance in livestock production and this fact is mainly represented by the emergence and spread of certain AMR and clones (livestock-associated) that reduce animal production [23].

Given the central role of livestock in understanding the genomic epidemiology of zoonotic staphylococci (especially, *S. aureus*) and the spread of AMR, the present study aims to determine the nasal *S. aureus*/MRSA carriage, antimicrobial resistomes and virulence determinants, genetic lineages, and immune evasion cluster (IEC) types among *S. aureus* isolates from pigs and pig-farmers. Also, the nasal carriage and species diversity of CoNS and co-carriage rate with *S. aureus* in the same nasal niche were studied.

## 2 Material and methods

### 2.1 Study Description and samples analyses

The study was performed in four pig farms (A-D) from the Aragon region (Spain), and were included 10 pigs from each farm (a total of 40 pigs) and 10 workers of the pig-farms (2, 3, 2 and 3 humans in farms A, B, C and D, respectively). Farm A had a total of 6000 piglets with an average weight of 20-22 kg and age of 9 weeks; Farm B had 15,000 piglets with an average weight of 9 kg and age range of 4–5 weeks; Farm C had 600 piglets with an average weight of 9 kg and age of 4–5 weeks; while Farm D had 400 piglets with an average weight of 10 kg and age of 6 weeks. All the pig-farmers had no pets in their houses, except one from farm A who had a dog and cat.

Nasal samples were collected (from January to March 2022) using sterile swabs with enrichment transport media (Amies). The ethical committee of the Universities of Zaragoza and La Rioja (Spain) reviewed and approved all procedures which were carried out following all applicable national, and/or international guidelines for human experiments (as described in the revised Helsinki declaration). Concerning the ethical use of animals, this study adhered to specific directives: 2010/63/EU and Spanish laws 9/2003 and 32/2007, RD 178/2004 and RD 1201/2005.

Samples were enriched in brain heart infusion broth (BHI; Condalab, Madrid, Spain) supplemented with 6.5% sodium chloride and incubated for 24 h at 37 °C. After overnight incubation, the broth samples were diluted and carefully dispensed onto four different bacteriological media: blood agar, mannitol salt agar (MSA; Condalab, Madrid, Spain), oxacillin resistance screening agar base (ORSAB with 2 mg/L oxacillin; Oxoid, Hampshire, UK) and ChromAgar LIN (Paris, France) and incubated for 24 h at 37 °C, for bacterial recovery. After overnight growth, 4 to 9 different colonies with staphylococci morphology were randomly selected per sample and identified by matrix-assisted laser desorption/ionization time-of-flight (MALDI-TOF; Bruker Daltonics, Bremen, Germany) following the standard extraction method as described by Bruker.

### 2.2 Antimicrobial susceptibility testing

The antimicrobial susceptibility of 12 different antimicrobial agents was performed by the disk diffusion method on all the recovered staphylococci following the recommendations and breakpoints of the European Committee on Antimicrobial Susceptibility Testing (EUCAST, 2022). The antimicrobial agents tested were as follows (μg/disk): penicillin (PEN) (1 or 10, depending on the staphylococci species), cefoxitin (FOX) (30), clindamycin (CLI) (2), erythromycin (ERY) (15), tobramycin (TOB) (10), gentamicin (GEN) (10), tetracycline (TET) (30), ciprofloxacin (CIP) (5), chloramphenicol (CLO) (30), linezolid (LZD) (10), mupirocin (MUP) (200), and trimethoprim-sulfamethoxazole (SXT) (1.25 + 23.75).

Once the antimicrobial resistance phenotype of all staphylococci was determined, isolates of different samples or those of the same sample but of different staphylococcal species and/or different AMR phenotypes were selected for further studies (considered as distinct isolates).

### 2.3 *S. aureus* DNA extraction

For DNA extraction, the isolates were seeded on BHI agar and incubated for 24 h at 37 °C. An isolated colony was suspended in 45 μL of sterile MiliQ water and later added 5 μL of lysostaphin (1 mg/mL) (Sigma®). The mixture was vortexed and incubated for 10 min at 37 °C. Forty-five μL of sterile MiliQ water, 150 μL of Tris-HCl (0.1 M, pH 8) and 5 μL of proteinase K (2 mg/mL) (Sigma®) were added. This was vortexed and incubated for 10 min at 60 °C. Finally, it was boiled for 5 min at 100 °C and centrifuged at 12,000 rpm for 3 min. The DNA samples were stored at −20 °C.

### 2.4 Mechanisms of antimicrobial resistance

The presence of the following resistance genes was tested by single PCRs, selected according to the antimicrobial resistance phenotype of isolates: beta-lactams (*mecA*), erythromycin and clindamycin (*ermA, ermB, ermC, ermT, mphC, msrA, lnuA,* and *lnuB*), aminoglycosides (*aac6’-aph2”,* and *ant4’*)*,* tetracycline (*tetL, tetM,* and *tetK)*, trimethoprim (*dfrA, dfrD, dfrG* and *dfrK*)*,* and chloramphenicol (*catpC221, catpC223, catpC194, catA, fexA,* and *fexB)* (Supplementary Table S1)*.* For the chloramphenicol-resistant isolates, they were PCR screened for the presence of the linezolid transferable resistance genes (*cfr, cfrB, cfrD, poxtA,* and *optrA*). Also, mutations in 23S-rDNA were screened by PCR/sequencing (Supplementary Table S1)*.*

### 2.5 Detection of virulence and IEC genes of *S. aureus* isolates

The presence of the *tst* and *luk-S/F-PV* virulence genes (encoding the toxin of shock toxic syndrome and Panton-Valentine leucocidin) was tested by PCR (Supplementary Table S1). The Immune Evasion Cluster (IEC) genes (*scn, chp, sak, sea,* and *sep*) were analysed and classified accordingly into 7 different IEC types (A to G), based on the combination of the positive genes. The *scn* gene was used as a biomarker of the presence of the IEC.

### 2.6 Molecular typing of isolates

All recovered *S. aureus* isolates were characterized by *spa* typing by PCR/Sanger sequencing. CC398 clone was determined by a specific PCR protocol for the *sau1*-*hsdS1* variant developed by Stegger et al [24]. The clonal complex of the isolates was assigned, when possible, according to the *spa*-types. Primers and conditions of PCRs performed in this study are included in Supplementary Table S1. Collections of positive control strains that contain AMR and virulence genes confirmed by sequencing at the Universidad de La Rioja were included in all the PCR assays in this study.

### 2.7 Statistical analyses

Data collected were verified and processed and the Statistical Package for Social Sciences (SPSS) Version 26 (IBM, California, U.S.A) was used for analysis. Data were reported as numbers and percentages (for categorical variables). Tables and charts were plotted. Data were subjected to univariate logistic regression to compute Odd Ratio (OR) at a 95% confidence interval (95%CI) of the association between the presence of MRSA, MSSA and the number of CoNS species in pigs and pig-farmers. A significant association was set <0.05 probability value.

## 3 Results

### 3.1 Nasal staphylococci diversity in healthy pigs and pig-farmers

A total of 243 staphylococci were isolated and identified from the nasal samples of healthy pigs and pig-farmers and they were distributed into 10 species. Of this, 142 *S. aureus,* 29 *S. sciuri*, 17 *S. haemolyticus*, 15 *S. chromogenes*, 13 *S. epidermidis,* 11 *S. hyicus*, 7 *S. saprophyticus*, 4 *S. simulans*, 3 *S. xylosus* and 2 *S. pasteuri* isolates were recovered from 38 nasal samples of pigs and 9 of pig-farmers (Table 1**).**

**Table 1:** Number of isolates and carriage rate of each staphylococci species recovered from the nasal samples of pigs and pig-farmers in four Spanish farms (A-D).

Species	N^o^ of isolates from pigs in farm A	N^o^ (%) of pigs from farm A	N^o^ of isolates from pigs in farm B	N^o^ (%) of pigs from farm B	N^o^ of isolates from pigs in farm C	N^o^ (%) of pigs from farm C	N^o^ of isolates from pigs in farm D	N^o^ (%) of pigs from farm D	N^o^ of isolates from pigs in all farms	No. (%) of pigs from all farms
*S. aureus*	18	6 (60)	31	7 (70)	14	3 (30)	43	10 (100)	106	26 (65)
*S. chromogenes*	9	5 (50)	1	1 (10)	2	2 (20)	1	1 (10)	13	9 (22.5)
*S. haemolyticus*	7	4 (40)	7	3 (30)	0	0 (0)	1	1 (10)	15	8 (20)
*S. hyicus*	3	3 (30)	5	3 (30)	2	2 (20)	0	0 (0)	10	8 (20)
*S. sciuri*	10	6 (60)	0	0 (0)	19	9 (90)	0	0 (0)	29	6 (15)
*S. epidermidis*	4	4 (40)	1	1 (10)	0	0 (0)	0	0 (0)	5	5 (12.5)
*S. saprophyticus*	5	2 (20)	1	1 (10)	0	0 (0)	0	0 (0)	6	3 (7.5)
*S. xylosus*	0	0 (0)	0	0 (0)	3	2 (20)	0	0 (0)	3	2 (5)
*S. pasteuri*	2	2 (20)	0	0 (0)	0	0 (0)	0	0 (0)	2	2 (5)
*S. simulans*	1	1 (10)	0	0 (0)	0	0 (0)	0	0 (0)	1	1 (2.5)


Species	N^o^ of isolates from pig-famers in farm A	N^o^ (%) of pig-famers from farm A	N^o^ of isolates from pig-famers in farm B	N^o^ (%) of pig-famers from farm B	N^o^ of isolates from pig-famers in farm C	N^o^ (%) of pig-famers from farm C	N^o^ of isolates from pig-famers in farm D	N^o^ (%) of pig-famers from farm D	N^o^ of isolates from pig-famers in all farms	No. (%) of pig-farmers from all farms
*S. aureus*	5	1 (50)	15	3 (100)	4	2 (100)	12	2 (66.7)	36	8 (80)
*S. chromogenes*	0	0 (0)	0	0 (0)	0	0 (0)	2	1 (33.3)	2	1 (10)
*S. haemolyticus*	0	0 (0)	0	0 (0)	0	0 (0)	2	1 (33.3)	2	1 (10)
*S. hyicus*	0	0 (0)	1	1 (33.3)	0	0 (0)	0	0 (0)	1	1 (10)
*S. sciuri*	0	0 (0)	0	0 (0)	0	0 (0)	0	0 (0)	0	0 (0)
*S. epidermidis*	5	1 (50)	2	2 (66.6)	1	1 (50)	0	0 (0)	8	4 (40)
*S. saprophyticus*	0	0 (0)	1	1 (33.3)	0	0 (0)	0	0 (0)	1	1 (10)
*S. xylosus*	0	0 (0)	0	0 (0)	0	0 (0)	0	0 (0)	0	0 (0)
*S. pasteuri*	0	0 (0)	0	0 (0)	0	0 (0)	0	0 (0)	0	0 (0)
*S. simulans*	0	0 (0)	0	0 (0)	1	1 (50)	2	1 (33.3)	3	2 (20)

***Note:*** Between 4 to 9 different staphylococci colonies were randomly selected per sample.

Concerning the nasal staphylococcal species in the pigs, 65% of the animals were *S. aureus* carriers, and the carriage rate for other species were: *S. chromogenes* (22.5%), *S. haemolyticus* (20%), *S. hyicus* (20%), *S. sciuri* (15%), *S. epidermidis* (12.5%), *S. saprophyticus* (7.5%), *S. xylosus* (5%), *S. pasteuri* (5%) and *S. simulans* (2.5%) (Table 1). Whereas, the nasal staphylococci carriage in pig-farmers was highest for *S. aureus* (80%), *S. epidermidis* (40%), *S. simulans* (20%), and 10% each for *S. chromogenes, S. saprophyicyus, S. hyicus* and *S. haemolyticus*. None of the pig-farmers had nasal carriage of *S. xylosus, S. sciuri* and *S. pasteuri* (Table 1**).**

### 3.2 Phenotypic and genetic characteristics of *S. aureus* isolates

**Fig. 1:**
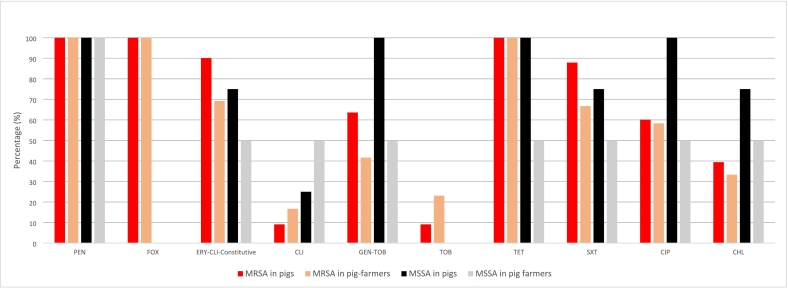
Antimicrobial resistance rates in *S. aureus* isolates from farms A to D (both pigs and farmers). Percentages were based on the collection of *S. aureus* obtained of different samples or those of the same sample but with different AMR phenotype (10, 16, 9, 16 from farms A to D, respectively). *Note:* There were 33 and 12 distinct MRSA isolates from pigs and pig-farmers respectively. Conversely, 4 and 2 distinct MSSA isolates from pigs and pig-farmers, respectively. CLO: chloramphenicol; CLI: clindamycin; CIP: ciprofloxacin; ERY: erythromycin; FOX: cefoxitin; GEN: gentamicin; PEN: penicillin; SXT: sulfamethoxazole/trimethoprim; TET: tetracycline, TOB: tobramycin.

After AMR phenotype determination of all the 142 *S. aureus* isolates, 51 distinct isolates were selected for further characterization that corresponded to one per sample or more than one if they showed different AMR phenotypes. Of all the 51 distinct *S. aureus* isolates, only 6 (11.8%, 4 from pigs and 2 from pig-farmers) were methicillin-susceptible (MSSA) and were all from farm-C. Essentially, the MRSA isolates from pigs (*n* = 33) harboured AMR as follows [percentage of resistant isolates/resistance genes detected]: penicillin [100], cefoxitin [100/*mecA*], erythromycin-clindamycin-constitutive [90.1/*ermB, ermC, ermT*], clindamycin [9.1/*lnuB*], gentamicin-tobramycin [63.6/*aac6’-aph2”*], tobramycin [9.1/*ant4’*], tetracycline [100/*tetK, tetL, tetM*], ciprofloxacin [60.1], sulfamethoxazole-trimethoprim [87.9/*dfrA, dfrG, dfrK*], and chloramphenicol [39.4/*fexA, catpC221*]. Moreover, the 12 distinct MRSA isolates from pig-farmers harboured AMR as follows: penicillin [100], cefoxitin [100/*mecA*], erythromycin-clindamycin-constitutive [69.2/*ermC, ermT*], clindamycin [16.7/*lnuB*], gentamicin-tobramycin [41.6/*aac6’-aph2”*], tobramycin [23.1/*ant4’*], tetracycline [100/*tetK, tetM*], ciprofloxacin [58.3], sulfamethoxazole-trimethoprim [66.7/*dfrA, dfrG, dfrK*], and chloramphenicol [25/*fexA, catpC221*] (Fig. 1 and Table 2).

**Table 2:** With-hosts and -farm variations of resistomes and genetic lineages of *S. aureus* isolates from all pigs and pig-farmers of the four analysed farms (A-D).

Farm	Host/ ID number	No. of isolates	AMR Phenotypes	AMR genes detected	*spa* type/CC a	IEC type
A	Pig 1	1	PEN-FOX-SXT-ERY-CLI-TET-CIP	*mecA, dfrK, ermC, tetK, tetM*	t011/CC398	Negative

	Pig 2	1	PEN-FOX-SXT-ERY-CLI-TET-TOB	*mecA, dfrA, ermB, ermC, tetM, ant4', aac6'-aph2''*	t011/CC398	Negative
	Pig 4	3	PEN-FOX-SXT-ERY-CLI-TET-CIP	*mecA, dfrK, ermC, ermT, tetK, tetL, tetM*	t011/CC398	Negative
	Pig 5	4	PEN-FOX-SXT-ERY-CLI-TET-TOB-GEN	*mecA, dfrK, ermC, tetM, aac6'-aph2''*	t011/CC398	Negative
	Pig 5	3	PEN-FOX-SXT-ERY-CLI-TOB-TET	*mecA, dfrA, ermC, tetK, tetM, ant4', aac6'-aph2''*	t011/CC398	Negative
	Pig 6	3	PEN-FOX-SXT-ERY-CLI-TET-TOB-GEN	*mecA, dfrA, ermB, ermC, tetM, aac6'-aph2''*	t011/CC398	Negative
	Pig 6	1	PEN-FOX-SXT-ERY-CLI-TET-TOB	*mecA, dfrA, ermB, ermC, tetK, tetM, ant4', aac6'-aph2''*	t011/CC398	Negative
	Pig 8	2	PEN-FOX-SXT-ERY-CLI-TET	*mecA, dfrG, ermA, tetM*	t4571/CC398	Negative
	Pig-farmer 2	2	PEN-FOX-SXT-ERY-CLI-TET-TOB	*mecA, dfrK, ermC, ermT, tetK, tetL, tetM*	t011/CC398	Negative
	Pig-farmer 2	3	PEN-FOX-SXT-ERY-CLI-TET-CIP-GEN-TOB	*mecA, dfrK, ermC, tetM, aac6'-aph2''*	t011/CC398	Negative

B	Pig 1	2	PEN-FOX-SXT-ERY-CLI-TET-TOB-GEN	*mecA, ermC, tetK, tetM, aac6'-aph2''*	t011/CC398	Negative

	Pig 1	1	PEN-FOX-SXT-ERY-CLI-TET-TOB-GEN-CLO	*mecA, dfrG, ermB, ermC, tetM, aiac6'-aph2'', fexA*	t011/CC398	Negative
	Pig 3	6	PEN-FOX-SXT-ERY-CLI-TET-TOB-GEN	*mecA, ermC, tetK, tetM, aac6'-aph2''*	t011/CC398	Negative
	Pig 4	3	PEN-FOX-SXT-ERY-CLI-TET-TOB-GEN	*mecA, dfrA, ermC, tetM, aac6'-aph2''*	t011/CC398	Negative
	Pig 5	3	PEN-FOX-SXT-ERY-CLI-TET-TOB-GEN	*mecA, ermC, tetK, tetM, aac6'-aph2''*	t011/CC398	Negative
	Pig 7	1	PEN-FOX-CLI-TET-TOB-GEN-CLO-CIP	*mecA, tetK, tetM, aac6'-aph2'', fexA*	t011/CC398	Negative
	Pig 7	1	PEN-FOX-CLI-TET-CIP	*mecA, tetK, tetM*	t011/CC398	Negative
	Pig 7	3	PEN-FOX-CLI-TET-TOB-GEN-CIP	*mecA, tetM, aac6'-aph2''*	t011/CC398	Negative
	Pig 8	4	PEN-FOX-SXT-ERY- CLI-TET-TOB-GEN	*mecA, dfrA, ermB, ermC, tetM, aac6'-aph2''*	t011/CC398	Negative
	Pig 9	7	PEN-FOX-SXT-ERY- CLI-TET-TOB-GEN	*mecA, dfrK, ermC, tetM, aac6'-aph2''*	t011/CC398	Negative
	Pig farmer 1	4	PEN-FOX-ERY-CLI-TET-TOB-CLO-CIP	*mecA, dfrG, ermC, lnuB, tetK, tetM, ant4', fexA*	t034/CC398	Negative
	Pig farmer 1	1	PEN-FOX-CLI-TET-TOB-CLO-CIP	*mecA, dfrG, lnuB, tetK, tetM, ant4', fexA*	t034/CC398	Negative
	Pig farmer 2	2	PEN-FOX-TET-CIP	*mecA, dfrA, tetM,*	t011/CC398	Negative
	Pig farmer 2	2	PEN-FOX-SXT-ERY-CLI-TET-TOB-GEN	*mecA, dfrK, ermC, tetM, aac6'-aph2''*	t011/CC398	Negative
	Pig farmer 3	5	PEN-FOX-SXT-ERY-CLI-TET-TOB-GEN	*mecA, dfrK, ermC, tetM, aac6'-aph2''*	t011/CC398	Negative
	Pig farmer 3	1	PEN-FOX-SXT-ERY-CLI-TET-TOB-GEN-CLO	*mecA, dfrK, ermC, tetK, tetM, aac6'-aph2'', ant4', fexA*	t011/CC398	Negative

C	Pig 1	1	PEN-SXT-CLI-TET-TOB-GEN-CIP	*dfrA, lnuB, tetK, tetM, aac6'-aph2''*	t191/CC9	Negative

	Pig 1	2	PEN-SXT-CLI-TET-TOB-GEN-CLO-CIP	*ermB, lnuB, tetL, tetM, aac6'-aph2'', fexA*	t1430/CC9	Negative
	Pig 3	5	PEN-SXT-CLI-TET-TOB-GEN-CLO-CIP	*dfrA, lnuB, ermB, ermC, tetK, tetM, aac6'-aph2'', fexA*	t1430/CC9	Negative
	Pig 5	3	PEN-FOX-SXT-ERY-CLI-TET-CIP	*mecA, dfrK, ermB, tetK, tetM*	t011/CC398	Negative
	Pig 5	2	PEN-FOX-ERY-CLI-TET-CIP	*mecA, ermB, tetK, tetM*	t011/CC398	Negative
	Pig 5	1	PEN-CLI-TET-TOB-GEN-CLO-CIP	*lnuB, tetK, tetL, aac6'-aph2'', fexA*	t1430/CC9	Negative
	Pig farmer 1	2	PEN-FOX-CLI-TET-CIP	*mecA, dfrA, ermC, lnuB, tetK, tetM*	t1451/CC398	Negative
	Pig farmer 1	1	PEN-SXT- CLI-TET-TOB-GEN-CLO-CIP	*dfrA, dfrG, tetK, tetM, aac6'-aph2'', fexA*	t1430/CC9	Negative
	Pig farmer 2	1	PEN	NT	t065/CC45	C

D	Pig 1	5	PEN-FOX-SXT-ERY-CLI-TET-TOB-GEN-CLO-CIP	*mecA, dfrK, ermC, tetK, tetM, catpC221*	t011/CC398	Negative

	Pig 2	5	PEN-FOX-SXT-ERY-CLI-TET-CLO-CIP	*mecA, dfrK, ermC, tetK, tetM, fexA*	t011/CC398	Negative
	Pig 2	1	PEN-FOX-SXT-ERY-CLI-TET-TOB-GEN-CLO-CIP	*mecA, dfrK, ermC, tetK, tetM, catpC221*	t011/CC398	Negative
	Pig 3	3	PEN-FOX-SXT-ERY-CLI-TET-TOB-GEN-CLO-CIP	*mecA, dfrK, ermC, tetK, tetM, catpC221*	t011/CC398	Negative
	Pig 4	3	PEN-FOX-SXT-ERY-CLI-TET-TOB-GEN-CLO-CIP	*mecA, dfrK, ermC, tetK, tetM, catpC221*	t011/CC398	Negative
	Pig 4	2	PEN-FOX-SXT-ERY-CLI-TET-CLO-CIP	*mecA, dfrK, ermC, tetK, tetM, fexA*	t011/CC398	Negative
	Pig 5	3	PEN-FOX-SXT-ERY-CLI-TET-TOB-GEN-CLO-CIP	*mecA, dfrK, ermC, tetK, tetM, catpC221*	t011/CC398	Negative
	Pig 6	4	PEN-FOX-SXT-ERY-CLI-TET-TOB-GEN-CLO-CIP	*mecA, dfrK, ermC, tetK, tetM, catpC221*	t011/CC398	Negative
	Pig 7	3	PEN-FOX-SXT-ERY-CLI-TET-TOB-GEN-CLO-CIP	*mecA, dfrK, ermC, tetK, tetM, catpC221*	t011/CC398	Negative
	Pig 7	2	PEN-FOX-SXT-ERY-CLI-TET-CIP	*mecA, dfrK, ermC, tetK, tetM*	t011/CC398	Negative
	Pig 8	6	PEN-FOX-SXT-ERY-CLI-TET-TOB-GEN-CLO-CIP	*mecA, dfrK, ermC, tetK, tetM, catpC221*	t011/CC398	Negative
	Pig 9	1	PEN-FOX-SXT-ERY-CLI-TET-TOB-GEN-CLO-CIP	*mecA, dfrK, ermC, tetK, tetM, catpC221*	t011/CC398	Negative
	Pig 10	5	PEN-FOX-SXT-ERY-CLI-TET-TOB-GEN-CLO-CIP	*mecA, dfrK, ermC, tetK, tetM, catpC221*	t011/CC398	Negative
	Pig farmer 1	5	PEN-FOX-SXT-ERY-CLI-TET-TOB-GEN-CLO-CIP	*mecA, dfrK, ermC, tetK, tetM, catpC221*	t011/CC398	Negative
	Pig farmer 1	2	PEN-FOX-SXT-ERY-CLI-TET-CIP	*mecA, dfrK, ermC, tetK, tetM*	t011/CC398	Negative
	Pig farmer 3	5	PEN-FOX-SXT-ERY-CLI-TET-CIP	*mecA, dfrK, ermC, tetK, tetM*	t011/CC398	Negative

CLO: chloramphenicol; CLI: clindamycin; CIP: ciprofloxacin; ERY: erythromycin; FOX: cefoxitin; GEN: gentamicin; PEN: penicillin; SXT: sulfamethoxazole/trimethoprim; TET: tetracycline, TOB: tobramycin.

*Note:* All isolates were *luk-S/F-PV* and *tst* negative.

a CC assigned according to the *spa-*type, except for CC398 (determined by specific PCR) NT: Not tested.

Regarding the 4 MSSA isolates from pigs, they harboured AMR as follows [percentage of resistance/detected genes]: penicillin [100], erythromycin-clindamycin-constitutive [75/*ermC*], clindamycin [25/*lnuB*], gentamicin-tobramycin [100/*aac6’-aph2”*], tetracycline [100/*tetK, tetM*], ciprofloxacin [100], trimethoprim-sulfamethoxazole [75/*dfrA, dfrK*] and chloramphenicol [75/*fexA*] (Fig. 1 and Table 2). However, one of the 2 MSSA from the pig-farmers was resistant to only penicillin, while the other harboured *dfrA, dfrG, tetK, tetM, aac6’-aph2”* and *fexA* resistance genes (Table 2).

### 3.3 Genetic typing of the *S. aureus* isolates from healthy pig and pig-farmers

**Fig. 2:**
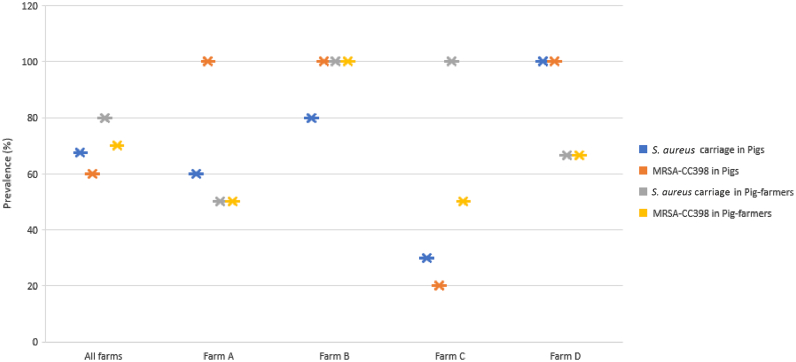
Frequency of *S. aureus* and MRSA-CC398 nasal carriage in pigs and pig-farmers.

All MRSA from pigs and pig-farmers were of the CC398 lineage. The prevalence of MRSA-CC398 lineage among the pigs studied was of 60%, while 70% of all the pig-farmers were MRSA-CC398 carriers (Fig. 2). Also, all MRSA isolates from farms A, B and D belonged to CC398 lineage, however, only 20% of the pigs from farm C carried MRSA-CC398 (Fig. 2). Based on the *spa-*types of the MRSA-CC398 isolates of pigs, all were t011, except one (which was t4571) (Table 2). However, of the 12 MRSA from the pig-farmers, MRSA-CC398-t011 (75%) was the predominant, followed by MRSA-CC398-t034 (16.7%), and then MRSA-CC398-t1451 (8.3%).

MSSA isolates were only detected from pigs and pig-farmers in farm C (66.7% of all isolates). The majority of the MSSA were of the CC9 lineage and *spa-*types t191 (*n* = 1) and t1430 (*n* = 7). Specifically, all the MSSA isolates from the pigs were MSSA-CC9, whereas, MSSA-CC45-t065 and MSSA-CC9-t1430 were identified from two pig-farmers (Table 2).

All the *S. aureus* isolates were negative for *luk-S/F-PV* and *tst* genes. All the *S. aureus* were *scn*-negative except one MSSA isolate from farm C that was *scn*-positive (IEC-type C) (Table 2).

### 3.4 Within-host variation of genetic lineages and/or AMR in pigs and pig-famers

Of the 26 pigs with nasal *S. aureus* carriage, 9 (34.6%) harboured isolates with varied within-host *spa*-types or resistomes (Table 2). Of these, 2 to 3 genetically distinct *S. aureus* isolates were detected (Table 2). In one of the pigs, one MSSA-CC9 and two MRSA-CC398 strains were detected (pig No. 5 in farm-C). The isolates also had different AMR phenotypes/genes, viz.: (PEN-FOX-SXT-ERY-CLI-TET-CIP/ *mecA, dfrK, ermB, tetK, tetM*; PEN-FOX-ERY-CLI-TET-CIP/ *mecA, ermB, tetK, tetM*; and PEN-CLI-TET-TOB-GEN-CLO-CIP/ *lnuB, tetK, tetL, aac6’-aph2”, fexA*). Also, worth mentioning is the detection in a single pig of an MSSA-CC9-t191 isolate carrying *dfrA, lnuB, tetK, tetM, aac6’-aph2”* genes and an MSSA-CC9-t1430 isolate carrying *ermB, lnuB, tetL, tetM, aac6’-aph2”, fexA* genes (Table 2).

Moreover, 6 (75%) of the pig-farmers had *S. aureus* isolates with varied within-host *spa*-types or AMR genes (Table 2). Of special relevance is the detection of an MSSA-CC9-t1430 with *dfrA, dfrG, tetK, tetM, aac6’-aph2”, fexA* resistance genes and an MRSA-CC398-t1451 with *mecA, dfrA, ermC, lnuB, tetK, tetM* genes (Table 2).

### 3.5 Nasal co-carriage of CoNS and *S. aureus* in pigs and pig-farmers

The majority of the hosts with co-carriage of single CoNS species with *S. aureus* were due to *S. chromogenes* and *S. haemolyticus* (Table 3). Nevertheless, most of the hosts with only *S. sciuri* carriage had no *S. aureus* co-carriage (especially in farm C) (Table 3). About 40% of pigs and pig-farmers with MRSA-CC398 had no other CoNS nasal co-carriage, whereas 36.7% had one CoNS co-carriage and 23.3% had ≥2 CoNS carriage (Table 4). Conversely, 16.7% of MSSA carriers had no CoNS co-carriage, whereas 33.3% had one CoNS co-carriage and 50% had ≥2 CoNS carriages (Table 4). About 41.1% who were not *S. aureus* carriers had ≥2 CoNS carriage (Table 4). However, there was no significant association between the presence of MRSA, MSSA and the number of CoNS species in pigs and pig-farmers (*p* > 0.05).

**Table 3:** Nasal staphylococci co-carriage in all pigs and pig farmers in the four analysed farms (A-D).

Farm	Host/ N^o^ of carriers	CoNS present	Presence of *S. aureus*	Methicillin Susceptibility/*spa* type/CC ^a^
A	Pig 1	*S. hyicus, S. simulans, S. epidermidis*	Yes	MRSA/t011/CC398

	Pig 2	*S. sciuri, S. haemolyticus, S. epidermidis*	Yes	MRSA/t011/CC398
	Pig 3	*S. chromogenes, S. sciuri*	No	NT
	Pig 4	*S. chromogenes*	Yes	MRSA/t011/CC398
	Pig 5	*S. hyicus*	Yes	MRSA/t011/CC398
	Pig 6	*S. hyicus*	Yes	MRSA/t011/CC398
	Pig 7	*S. haemolyticus, S. epidermidis, S. chromogenes, S. saprophyticus*	No	NT
	Pig 8	*S. chromogenes, S. haemolyticus, S. pasteuri*	Yes	MRSA/ t4571/CC398
	Pig 9	*S. haemolyticus, S. sciuri*	No	NT
	Pig 10	*S. pasteuri, S. chromogenes, S. saprophyticus*	No	NT
	Pig-farmer 1	*S. epidermidis*	No	NT
	Pig-farmer 2	None	Yes	MRSA/t011/CC398

B	Pig 1	*S. epidermidis, S. haemolyticus, S, hyicus*	Yes	MRSA/t011/CC398

	Pig 2	*S. hyicus*	No	NT
	Pig 3	*S. hyicus*	Yes	MRSA/t011/CC398
	Pig 4	*S. haemolyticus*	Yes	MRSA/t011/CC398
	Pig 5	*S. haemolyticus*	Yes	MRSA/t011/CC398
	Pig 6	None	No	NT
	Pig 7	None	Yes	MRSA/t011/CC398
	Pig 8	None	Yes	MRSA/t011/CC398
	Pig 9	*S. chromogenes*	Yes	MRSA/t011/CC398
	Pig 10	None	No	NT
	Pig farmer 1	*S. hyicus, S. epidermidis, S. saprophyticus*	Yes	MRSA/t034/CC398
	Pig farmer 2	*S. epidermidis*	Yes	MRSA/t034/CC398
	Pig farmer 3	None	No	NT

C	Pig 1	*S. sciuri, S. chromogenes, S. hyicus*	Yes	MSSA/t191/CC9; MSSA/t1430/CC9

	Pig 2	*S. sciuri*	No	NT
	Pig 3	*S. sciuri*	Yes	MSSA/t1430/CC9
	Pig 4	*S. sciuri*	No	NT
	Pig 5	*S. chromogenes*	Yes	MRSA/t011/CC398; MSSA/t1430/CC9
	Pig 6	*S. sciuri*	No	NT
	Pig 7	*S. sciuri*	No	NT
	Pig 8	*S. sciuri*	No	NT
	Pig 9	*S. hyicus. S. xylosus*	No	NT
	Pig 10	*S. xylosus, S. sciuri*	No	NT
	Pig farmer 1	*S. epidermidis, S. simulans*	Yes	MRSA/t1451/CC398; MSSA/t1430/CC9
	Pig farmer 2	None	Yes	MSSA/t065/CC45

D	Pig 1	*S. chromogenes*	Yes	MRSA/t011/CC398

	Pig 2	None	Yes	MRSA/t011/CC398
	Pig 3	None	Yes	MRSA/t011/CC398
	Pig 4	None	Yes	MRSA/t011/CC398
	Pig 5	*S. haemolyticus*	Yes	MRSA/t011/CC398
	Pig 6	None	Yes	MRSA/t011/CC398
	Pig 7	None	Yes	MRSA/t011/CC398
	Pig 8	None	Yes	MRSA/t011/CC398
	Pig 9	None	Yes	MRSA/t011/CC398
	Pig 10	None	Yes	MRSA/t011/CC398
	Pig farmer 1	None	Yes	MRSA/t011/CC398
	Pig farmer 2	*S. simulans, S. haemolyticus*	No	NT
	Pig farmer 3	*S. chromogenes*	Yes	MRSA/t011/CC398

NT: Not tested.

*Note:*^a^ CC assigned according to the *spa-*type, except for CC398 (determined by specific PCR).

**Table 4:** Comparison matrix of the presence or absence of MRSA and MSSA isolates and the number of CoNS species in pigs and pig-farmers.

Pigs or pig farmers with:	No. (%) of pigs and farmers without CoNS	OR (95% CI)	*p* value	No. (%) of pigs and farmers with 1 CoNS species	OR (95% CI)	*p* value	No. (%) of pigs and farmers with ≥2 CoNS species	OR (95% CI)	*p* value
MRSA-CC398 (n = 30)	12 (40.0)	3.11 (0.73–13.2)	0.124	11 (36.7)	0.83 (0.24–2.79)	0.760	7 (23.3)	0.43 (0.12–1.57)	0.204
MSSA (n = 6)	1 (16.7)	0.93 (0.08–11.2)	0.956	2 (33.3)	0.71 (0.10–5.04)	0.736	3 (50.0)	1.43 (0.22–9.26)	0.708
Absence of *S. aureus* (n = 17)	3 (17.6)	Referent	Referent	7 (41.1)	Referent	Referent	7 (41.1)	Referent	Referent

Significant association determined by bivariate regression at 95% Confidence interval (CI).

*Note:* A pig and pig-farmer each had both MRSA-CC398 and MSSA-CC9 co-carriage. Also, 1 pig farmer had two MSSA-CC9 with different *spa* types (see Table 2).

## 4 Discussion

Several studies have reported the nasal carriage rates and transmission patterns of *S. aureus* between pigs and pig-farmers. Worth mentioning is that our research group have detected in the last decade the presence of MRSA, especially the CC398 in pigs, humans in-contact with pigs, pig-derived foods, pig-farm environmental samples and human residents close to pig farms as well as patients in hospitals located in areas with high pig density in Spain [21,[25], [26], [27], [28], [29], [30]]. These put together highlight the endemic status of MRSA-CC398 in Spain. However, the present study further elucidated the within-host variability of AMR of *S. aureus* of the same or different genetic lineages and their potential association with CoNS species in the same nasal niche. This information can better explain the complex existence of varied *spa-*types and AMR within the same CCs and their potential implication in the control of AMR in pig herds and zoonotic transmission.

Our findings showed that the most prevalent staphylococcal species in pigs was *S. aureus*. This is not unexpected as it is consistent with previous findings from similar designs in Spain which reported up to 89.6% carriage *S aureus* rate by Abreu et al. [31], 85.6% by Morcillo et al. [32] and other European countries such as 96.1% in Portugal by Lopes et al. [33]; 65.5% in Belgium by Peeters et al. [34]. Also, similar nasal *S. aureus* carriage rates in healthy pigs (75.2%) were reported in Australia [35] and the USA (67.7%) [36], India (71.4%) [37] and China (47.9%) [38]. However, lower frequencies were reported in a Spanish study, 12.7% [39] and in other countries in Africa and middle east Asia [40,41]. The varied frequencies of *S. aureus* detection rate reported by these studies could be due to the age of the pigs studied or variations in studied methodologies/protocols and the level of intensive pig-farming in the study areas [42].

Conversely, other CoNS detected in high frequencies among the pigs, such as *S. chromogenes* and *S. haemolyticus* corroborated with previous reports on the nasal CoNS carriage rate in livestock [40,43,44]. Although *S. sciuri* was reported in low rates from the pigs, a much higher prevalence of 80% was detected in healthy pigs in Ghana [40]. The low detection rate of *S. sciuri* from the pigs in our study and its absence in the pig-farmers could be due to the displacement of this species from the nasal cavity by *S. aureus,* as the individuals were heavily colonized by *S. aureus* [43]. However, this observation needs to be further elucidated.

Concerning the MRSA recovery rate in pigs, the majority of the *S. aureus* isolates (all in farms A, B, and D and few in farm C) were MRSA (>90%). This finding is similar to the previous report from another Spanish region (Catalonia) where all the *S. aureus* (100%) were methicillin-resistant [45]. Similarly, about 80% of the *S. aureus* from the pig-farmers were MRSA. However, this observation is different from another Spanish study in the Canary Islands, where a relatively low prevalence (15%) of nasal MRSA was reported in pig-farmers [32].

It has been shown that exposure to high amounts of MRSA in the environment (such as the air) of pig-farm and time spent on the farm are major important determinants for MRSA nasal carriage in pig-farmers [46]. Also, a higher pig density of farms could contribute to the nasal carriage rate of MRSA-CC398 in pig-farmers [45]. This could be the reason why MRSA-CC398 was relatively less in farm-C which had the least population of pigs.

The prevalence of MRSA found in pigs (62.5%) was similar to those reported in Germany (52%) and the Netherlands (56%) [46,47]. But much higher than the report from La Rioja (Spain), where a 21% MRSA nasal carriage rate was reported among fattening in a slaughterhouse [27]. These differences reflect the physical conditions and the age of the pigs during sampling collection.

A very interesting finding related to the MRSA-CC398 detected in the pig-farmers is the *spa-*type t034 and t1451 which was not detected in any of the pigs. Also, all the *S. aureus* isolates were IEC-negative (i.e., lacked the human-adaptation marker), except one MSSA-CC45-t065 from a pig-farmer which was IEC-type C. These put together suggest that the MRSA-CC398-t034 and -t1451 lineage and MSSA-CC9 from pig-farmers were animal-adapted subclades [48]. However, none of the pigs tested had MRSA-CC398 with these *spa-*types. This raises a question of the source of these MRSA-CC398-*spa-*types t034 and -t1451 isolates in the pig-farmers. Nevertheless, their absence, even in very low frequency cannot be categorically exonerated from the pig population.

Concerning the AMR phenotypes of the *S. aureus* isolates, all the MRSA-CC398 isolates showed tetracycline resistance. It has been demonstrated that tetracycline resistance is a good phenotypic marker of MRSA-CC398 [25,26,49] and the *tetM* gene is classically integrated into the SCC*mec* of MRSA-CC398 [45]. The MRSA isolates from this study showed high-level resistance to erythromycin and clindamycin. In 90.1% of the MRSA isolates from pigs, erythromycin-clindamycin constitutive resistance was detected (mediated mainly by *ermB* and *ermC*), while a small proportion showed solely clindamycin-resistance (with erythromycin susceptibility) mediated by the *lnuB* gene, which is often enriched among MRSA-CC398 isolates [28]. Importantly, the presence of *lnuA* or *lnuB* genes seems to be related to *S. aureus* animal-dependent lineages [50]. Regarding the MLS_B_ resistance genes, *ermT* was also detected in two strains from a pig and pig-farmer with similar AMR profiles. The *ermT* gene is very unusual in MRSA-CC398 isolates, in most cases, this gene (*ermT*) is associated with plasmids and metal resistance genes such as *cadD, cadX* and *copA* [51].

Of note, some of the pigs and pig-farmers had within-host diversity of genetic lineages and methicillin resistance profile (i.e., carriers of both MRSA-CC398 and MSSA-CC9). Also, heterogeneity in the AMR phenotypes and genes of within-host MRSA isolates was recorded in a significant number of pigs and pig-farmers with each host harbouring 2 or 3 distinct AMR phenotypes. These phenomena highlight the importance of selecting multiple colonies from all *S. aureus* nasal carriers to obtain complete epidemiological data.

It is important to mention the detection of some AMR genes that are often plasmid-mediated (*tetL*, *fexA*, *dfrG*) [52] and transposon-encoded (*tetM*, *dfrK*) [53]. This could denote selective pressure that might be responsible for their maintenance in the pigs and pig-farmers. Especially for the CLO^R^-*S. aureus* isolates since this antibiotic is no longer in use in pig-farming at the time of sample collection due to the new EU law that bans its use in animal husbandry [54]. In all of the MRSA and MSSA isolates (except one from a pig-farmer), a wide multidrug resistance phenotype with large arrays of resistance genes was detected. This is a classical characteristic of most MRSA and some MSSA isolates from pig-farm settings [28,45].

Concerning the co-carriage of *S. aureus* and CoNS, we did not find any statistical association between *S. aureus* carriage rate and other CoNS, so these findings could not be confirmed in the pigs and pig-farmers isolates. Perhaps, colonization with *S. aureus* can be associated with other bacterial species [14]. This study is not without a limitation. Importantly, insights into the genomic contents of methicillin-resistant-CoNS and how they modulate and could potentially serve as reservoirs for horizontal transmission of AMR genes to *S. aureus* are needful at the pig-farm level.

## 5 Conclusion

The very high level of MRSA with multidrug resistance phenotypes, within-host resistomes and genetic lineage diversities highlight the need for an enhanced multiple sampling to account for the evolution and dynamics of AMR crisis and control of inter-host transmissions of *S. aureus* in pig-farms using the “One Health” approach. Also, comparative genetic analysis of MRSA-CC398 and CoNS could help to elucidate the complex interactions of staphylococci and the flow of resistomes within the nasal niche.

## Funding

This work was supported by MCIN/AEI/10.13039/501100011033 (project PID2019-106158RB-I00) of Spain. Also, it received funding from the European Union’s H2020 research and innovation programme under the Marie Sklodowska-Curie grant agreement N° 801586.

## CRediT authorship contribution statement

**Idris Nasir Abdullahi:** Conceptualization, Methodology, Validation, Formal analysis, Data curation, Funding acquisition, Investigation, Software, Writing - original draft, Writing - review & editing. **Carmen Lozano:** Validation, Supervision, Data curation, Formal analysis, Writing - original draft, Writing - review & editing. **Carmen Simon:** Methodology, Formal analysis, Writing - original draft, Writing - review & editing. **Javier Latorre-Fernandez:** Methodology, Formal analysis, Writing - original draft, Writing - review & editing. **Myriam Zarazaga:** Validation, Formal analysis, Funding acquisition, Writing - original draft, Writing - review & editing. **Carmen Torres:** Conceptualization, Validation, Formal analysis, Data curation, Supervision, Project administration, Funding acquisition, Writing - original draft, Writing - review & editing.

## Declaration of Competing Interest

No conflict of interest declared.

## Data Availability

The data generated from this study have been thoroughly presented. However, further enquiries can be made through the corresponding author (C.T.).

## Appendix A Supplementary data

Supplementary material: Table S1 Image 1
