# Supplementary material for: Nasal staphylococci community of healthy pigs and pig-farmers in Aragon (Spain). Predominance and within-host resistome diversity in MRSA-CC398 and MSSA-CC9 lineages
Source: One Health. 2023 Feb 11;16:100505. doi: 10.1016/j.onehlt.2023.100505 (PMC10288040; doi:10.1016/j.onehlt.2023.100505)
Supplement: Supplementary file 1 — Supplementary material: Table S1 [file mmc1.docx]

**Table S1**. Genes and primers’ sequences utilized for all PCRs in this study

| **Gene** | **Primers’ oligonucleotide (5’**⇒**3’)** | **Amplicon size** | **Reference** |
| --- | --- | --- | --- |
| **Antimicrobial resistance (AMR) genes** | | | |
| *blaZ* | F: CAGTTCACATGCCAAAGAG | 772 bp | [1] |
|  | R: TACACTCTTGGCGGTTTC |  |  |
| *mecA* | F: GGGATCATAGCGTCATTATTC | 527 bp | [2] |
|  | R: AACGATTGTGACACGATAGCC |  |  |
| *ermA* | F: TCTAAAAAGCATGTAAAAGAA | 645 bp | [3] |
|  | R: CTTCGATAGTTTATTAATATTAG |  |  |
| *ermB* | F: GAAAAGTACTCAACCAAATA | 639 bp | [3] |
|  | R: AGTAACGGTACTTAAATTGTTTA |  |  |
| *ermC* | F: TCAAAACATAATATAGATAAA | 642 bp | [3] |
|  | R: GCTAATATTGTTTAAATCGTCAAT |  |  |
| *ermT* | F: CCGCCATTGAAATAGATCCT | 200 bp | [8] |
|  | R: TTCTGTAGCTGTGCTTTCAAAAA |  |  |
| *lnuA* | F: GGTGGCTGGGGGGTAGATGTATTAACTGG | 323 bp | [4] |
|  | R: GCTTCTTTTGAAATACATGGTATTTTTCGATC |  |  |
| *lnuB* | F: CCTACCTATTGTTTGTGGAA | 499 bp | [5] |
|  | R: ATAACGTTACTCTCCTATTC |  |  |
| *aac6’-aph2’’* | F: CCAAGAGCAATAAGGGCATA | 220 bp | [6] |
|  | R: CACTATCATAACCACTACCG |  |  |
| *ant4’* | F: GCAAGGACCGACAACATTTC | 165 bp | [6] |
|  | R: TGGCACAGATGGTCATAACC |  |  |
| *tetL* | F: CATTTGGTCTTATTGGATCG | 456 bp | [7] |
|  | R: ATTACACTTCCGATTTCGG |  |  |
| *tetM* | F: GTTAAATAGTGTTCTTGGAG | 576 bp | [7] |
|  | R: CTAAGATATGGCTCTAACAA |  |  |
| *tetK* | F: TTAGGTGAAGGGTTAGGTCC | 697 bp | [7] |
|  | R: GCAAACTCATTCCAGAAGCA |  |  |
| *dfrA* | F: CCTTGGCACTTACCAAATG | 374 bp | [1] |
|  | R: CTGAAGATTCGACTTCCC |  |  |
| *dfrD* | F: TTCTTTAATTGTTGCGATGG | 582 bp | [1] |
|  | R: TTAACGAATTCTCTCATATATATG |  |  |
| *dfrG* | F: TCGGAAGAGCCTTACCTGACAGAA | 323 bp | [8] |
|  | R: CCCTTTTTGGGCAAATACCTCATTCCA |  |  |
| *dfrK* | F: GAGAATCCCAGAGGATTGGG | 423 bp | [8] |
|  | R: CAAGAAGCTTTTCGCTCATAAA |  |  |
| *cat_pC221_* | F: ATTTATGCAATTATGGAAGTTG | 434 bp | [1] |
|  | R: TGAAGCATGGTAACCATCAC |  |  |
| *cat_pC223_* | F: GAATCAAATGCTAGTTTTAACTC | 283 bp | [1] |
|  | R: ACATGGTAACCATCACATAC |  |  |
| *cat_pC194_* | F: CGACTTTTAGTATAACCACAGA | 570 bp | [1] |
|  | R: GCCAGTCATTAGGCCTAT |  |  |
| *catA* | F: GGATATGAAATTTATCCCTC | 505 bp | [7] |
|  | R: CAATCATCTACCCTATGAAT |  |  |
| *fexA* | F: GTACTTGTAGGTGCAATTACGGCTGA | 1272 bp | [9] |
|  | R: CGCATCTGAGTAGGACATAGCGTC |  |  |
| *fexB* | F: TTCCCACTATTGGTGAAAGGAT | 816 bp | [10] |
|  | R: GCAATTCCCTTTTATGGACGTT |  |  |
| *cfr* | F: TGAAGTATAAAGCAGGTTGGGAGTCA | 746 bp | [11] |
|  | R: ACCATATAATTGACCACAAGCAGC |  |  |
| *cfrB* | F: TGAGCATATACGAGTAACCTCAAGA | 293 bp | [12] |
|  | R: CGCAAGCAGCGTCTATATCA |  |  |
| *cfrD* | F: AGAAGTCGCAACAAGTGAGGA | 595 bp | [13] |
|  | R: GCAACTGCATGAGTCAAAGAA |  |  |
| *optrA* | F: AGGTGGTCAGCGAACTAA | 1395 bp | [14] |
|  | R: ATCAACTGTTCCCATTCA |  |  |
| *poxtA* | F: TCAATGCAGAGCAGGAAGCA | 791 bp | [13] |
|  | R: GGTGGATTTACCGACACCGT |  |  |
| *23S-rDNA* | F: GCGGTCGCCTCCTAAAAG | 420 bp | [15] |
|  | R: ATCCCGGTCCTCTCGTACT |  |  |
| **IEC and virulence genes** | | | |
| *scn* | F: AGCACAAGCTTGCCAACATCG | 257 bp | [16] |
|  | R: TTAATATTTACTTTTTAGTGC |  |  |
| *chp* | F: TTTACTTTTGAACCGTTTCCTAC | 366 bp | [16] |
|  | R: CGTCCTGAATTCTTAGTATGCATATTCATTAG |  |  |
| *sak* | F: AAGGCGATGACGCGAGTTAT | 223 bp | [16] |
|  | R: GCGCTTGGATCTAATTCAAC |  |  |
| *sea* | F: AGATCATTCGTGGTATAACG | 344 bp | [16] |
|  | R: TTAACCGAAGGTTCTGTAGA |  |  |
| *sep* | F: AATCATAACCAACCGAATCA | 196 bp | [16] |
|  | R: TCATAATGGAAGTGCTATAA |  |  |
| *tst* | F: TTCACTATTTGTAAAAGTGTCAGACCCACT | 180 bp | [17] |
|  | R: TACTAATGAATTTTTTTATCGTAAGCCCTT |  |  |
| *lukS/F*-PV | F: ATCATTAGGTAAAATGTCTGGACATGATCCA | 443 bp | [18] |
|  | R: GCATCAAGTGTATTGGATAGCAAAAGC |  |  |
|  | R: AGTGAACTTATCTTTCTATTGAAAAACACTC |  |  |
| **Staphylococcal Protein A typing** | | | |
| *spa* | F: AGACGATCCTTCGGTGAGC | Hypervariable | [19] |
|  | R: GCTTTTGCAATGTCATTTACTG |  |  |

**References**

1. Schnellmann C, Gerber V, Rossano, A., Jaquier, V., Panchaud, Y., Doherr, M.G., Thomann, A., Straub, R., Perreten, V., 2006. Presence of new *mecA* and *mph*(C) variants conferring antibiotic resistance in *Staphylococcus* spp. isolated from the skin of horses before and after clinic admission. J. Clin. Microbiol. 44, 4444–4454.
2. Poulsen AB, Skov R, Pallesen LV. Detection of methicillin resistance in coagulase-negative staphylococci and in staphylococci directly from simulated blood cultures using the EVIGENE MRSA Detection Kit. J. Antimicrob Chemother 2013; 51, 419–421.
3. Sutcliffe J, Grebe T, Tait-Kamradt A, Wondrack L. Detection of erythromycin-resistant determinants by PCR. Antimicrob Agents Chemother 1996; 40, 2562–2566.
4. Lina G, Quaglia A, Reverdy ME, Leclercq R, Vandenesch F, Etienne J. Distribution of genes encoding resistance to macrolides, lincosamides, and streptogramins among staphylococci. Antimicrob Agents Chemother 1999; 43, 1062–1066.
5. Bozdogan B, Berrezouga L, Kou MS, Yurek DA, Farley KA, Stockman BJ, Leclercq R. A new resistance gene, *linB*, conferring resistance to lincosamides by nucleotidylation in *Enterococcus faecium* HM1025. Antimicrob Agents Chemother 1999; 43, 925–929.
6. van de Klundert J, Vliegenthart J. PCR detection of genes coding for aminoglycoside-modifying enzymes, in: Diagnostic Molecular Microbiology. Principles and Applications. 1993; pp. 547– 552. https://doi.org/10.1023/A:1016601629518
7. Aarestrup FM, Agerso Y, Gerner-Smidt P, Madsen M, Jensen LB. Comparison of antimicrobial resistance phenotypes and resistance genes in *Enterococcus faecalis* and *Enterococcus faecium* from humans in the community, broilers, and pigs in Denmark. Diagn Microbiol Infect 2000; Dis. 37, 127–137.
8. Gómez-Sanz E, Torres C, Lozano C, Fernández-Pérez R, Aspiroz C, Ruiz-Larrea F, Zarazaga M. Detection, molecular characterization, and clonal diversity of methicillin-resistant *Staphylococcus aureus* CC398 and CC97 in Spanish slaughter pigs of different age groups. Foodborne Pathog Dis 2010; 7, 1269–1277
9. Kehrenberg C, Schwarz S. Florfenicol-chloramphenicol exporter gene *fexA* is part of the novel transposon Tn*558*. Antimicrob. Agents Chemother 2005; 49, 813–815.
10. Liu H, Wang Y, Wu C, Schwarz S, Shen Z, Jeon B, Ding S, Zhang Q, Shen J. A novel phenicol exporter gene, *fexB*, found in enterococci of animal origin. J Antimicrob Chemother 2012; 67, 322–325.
11. Kehrenberg C, Schwarz S. Distribution of florfenicol resistance genes *fexA* and *cfr* among chloramphenicol-resistant *Staphylococcus* isolates. Antimicrob. Agents Chemother 2006; 50, 1156–1163.
12. Lee SM, Huh HJ, Song DJ, Shim HJ, Park KS, Kang CI, Ki CS, Lee NY. Resistance mechanisms of linezolid-non-susceptible enterococci in Korea: Low rate of 23S rRNA mutations in *Enterococcus faecium*. J. Med. Microbiol 2017; 66, 1730–1735
13. Ruiz-Ripa L, Feßler AT, Hanke D, Eichhorn I, Azcona-Gutiérrez JM, Pérez-Moreno MO, Seral C, Aspiroz C, Alonso CA, Torres L, Alós JI, Schwarz S, Torres C. Mechanisms of Linezolid Resistance Among Enterococci of Clinical Origin in Spain-Detection of *optrA*- and *cfr*(D)-Carrying *E. faecalis*. *Microorganisms*, 2020; *8*(8), 1155. https://doi.org/10.3390/microorganisms8081155
14. Wang Y, Lv Y, Cai J, Schwarz S, Cui L, Hu Z, et al. A novel gene, *optrA*, that confers transferable resistance to oxazolidinones and phenicols and its presence in *Enterococcus faecalis* and *Enterococcus faecium* of human and animal origin. J Antimicrob Chemother 2015; 70, 2182–2190.
15. Dibo I, Pillai SK, Gold HS, Baer MR, Wetzler M, Slack JL, et al. Linezolid-resistant *Enterococcus faecalis* isolated from a cord blood transplant recipient. J Clin Microbiol 2004; 42, 1843–1845.
16. Van Wamel WJB, Rooijakkers SHM, Ruyken M, van Kessel KPM, van Strijp JAG. The innate immune modulators staphylococcal complement inhibitor and chemotaxis inhibitory protein of *Staphylococcus aureus* are located on beta-hemolysin-converting bacteriophages. J Bacteriol 2006;188, 1310–1315.
17. Yamaguchi T, Nishifuji K, Sasaki M, Fudaba Y, Aepfelbacher M, Takata T, Ohara M, Komatsuzawa H, Amagai M, Sugai M. Identification of the *Staphylococcus aureus etd* pathogenicity island which encodes a novel exfoliative toxin, ETD, and EDIN-B. Infect Immun 2002; 70, 5835–5845.
18. Lina G, Quaglia A, Reverdy ME, Leclercq R, Vandenesch F, Etienne J. Distribution of genes encoding resistance to macrolides, lincosamides, and streptogramins among staphylococci. Antimicrob Agents Chemother 1999; 43, 1062–1066.
19. Harmsen D, Claus H, Witte W, Claus H, Turnwald D, Vogel U. Typing of methicillin-resistant *Staphylococcus aureus* in a university hospital setting by using novel software for *spa* repeat determination and database management. J Clin Microbiol 2003; 41, 5442–5448.
